# The Effect of *In Vitro* Digestion on Matcha Tea *(Camellia sinensis)* Active Components and Antioxidant Activity

**DOI:** 10.3390/antiox11050889

**Published:** 2022-04-30

**Authors:** Tereza Koláčková, Daniela Sumczynski, Antonín Minařík, Erkan Yalçin, Jana Orsavová

**Affiliations:** 1 Department of Food Analysis and Chemistry, Tomas Bata University in Zlín, Nám. T.G. Masaryka 1279, 76001 Zlín, Czech Republic; kolackova@utb.cz; 2 Department of Physics and Materials Engineering, Tomas Bata University in Zlín, Vavrečkova 275, 76001 Zlín, Czech Republic; minarik@utb.cz; 3 Department of Food Engineering, Gölköy Campus, Bolu Abant Ízzet Baysal University, Bolu 14030, Turkey; yalcin_e@ibu.edu.tr; 4 Language Centre, Tomas Bata University in Zlín, Štefánikova 5670, 76001 Zlín, Czech Republic; orsavova@utb.cz

**Keywords:** antioxidant activity, flavonoid, *in vitro* digestion, matcha tea, xanthine alkaloid, phenolic acid

## Abstract

This study investigates the effects of *in vitro* digestion on the antioxidant activity and release of phenolics, xanthine alkaloids, and L-theanine contents of matcha. It establishes digestibility values between 61.2–65.8%. Considering native matcha, the rutin content (303–479 µg/g) reached higher values than catechin (10.2–23.1 µg/g). Chlorogenic acid (2090–2460 µg/g) was determined as predominant. Rutin, quercetin, ferulic, ellagic, and caffeic acid were the least-released phenolics, and their remaining residues reached 76–84%. Protocatechuic, hydroxybenzoic acid, epigallocatechin, and epigallocatechin-3-gallate were the best-released phenolics, with the remaining residues under 1%. Caffeine, L-theanine, and theobromine contents in native matcha reached 16.1, 9.85, and 0.27 mg/g, respectively. Only caffeine (3.66–5.26 mg/g) and L-theanine (0.09–0.15 mg/g) were monitored in the undigested residue, representing 13 and 0.1% of the remaining part, respectively. A chemiluminescence assay showed that water-soluble antioxidants showed significant antioxidant activity in native matcha, while lipid-soluble compounds showed higher antioxidant activity in the undigested samples. Cinnamic and neochlorogenic acids were determined as the main contributors to the ACW values in the undigested matcha, epicatechin, and quercetin in the ACL fraction. The application of the digestion process reduced the antioxidant activity by more than 94%. SEM has proved specific digestion patterns of *in vitro* digestibility of matcha.

## 1. Introduction

Leaves from *Camellia sinensis* L. are widely used in the tea-making process [1,2]. Green tea, which provides many physiological advantages, including anti-inflammatory, antibacterial, and cholesterol-lowering advantages, may help decrease the risk of oxidative stress and cardiovascular diseases and may stimulate detoxification processes. Apart from these pharmacological properties, its antioxidant and antimutagenic effects have also contributed to the increase in its popularity [3,4,5,6,7]. Matcha is a powdered form of green tea leaves produced by grinding with a millstone. It is made from young shoots that have been covered for a few weeks in order to enrich them with higher concentrations of L-theanine and antioxidants predominantly from the group of phenolics and alkaloids [2,8,9,10]. The green tea contains a complex of polyphenols, alkaloids, free amino acids, proteins, vitamin C, organic acids, saponins, volatile compounds, minerals, and trace elements [3,4,5,9]. Xanthine alkaloids (caffeine: 1,3,7-trimethylxanthine; theobromine: 3,7-dimethylxanthine; theophylline: 1,3-dimethylxanthine) and L-theanine exhibit various beneficial physiological effects, including the stimulation of the central nervous system and the cardiovascular, respiratory, gastrointestinal, and renal systems and memory function improvement. Their concentrations depend on the plant genotype and affect the flavor of foods and beverages [11,12,13,14].

Polyphenols (especially catechins), caffeine and L-theanine occurring in green teas, maintain their high antioxidant activity while they are leached into water at temperatures above 60–80 °C. The precise content of tea leaf constituents depends on the tea type, its quantity, temperature, and time of brewing [2,12].

To the best of our knowledge, no studies have dealt with the cold brewing of matcha teas associated with polyphenol and alkaloid leaching. Matcha is consumed directly in the form of powder added to food as a valuable source of phenolics. The release of individual substances from food matrices determines their absorption behavior in the intestinal system. Therefore, the intake of food rich in polyphenols does not necessarily mean more phenolic compounds will be absorbed in the digestive tract. The interactions between biologically active substances and other components of the food matrix limit their complete release during gastrointestinal digestion. Hence, it is important to quantify the proportion of digestible or indigestible antioxidants which might be available for the gastrointestinal tract. The quantity of digested substances released during digestion and their availability for absorption by the intestinal brush border of the cells is expressed as their bioaccessibility. It is a key factor influencing further processes of ingested compounds, including their bioavailability and biological activity. It refers to the quantity of antioxidants which have passed though the cell membrane and are available for use within the cell. It also determines the stability of antioxidants throughout the process and monitors the concentrations of antioxidants released into the targeted cell to show their biological activity. The bioaccessibility index is the prerequisite for bioactive substances to express their effects on human health. Since animal or human trials are labor intensive and ethically disputable, *in vitro* digestion models have been widely established and employed for the investigation of changes in the phytochemical profile during digestion processes and the prediction of their bioaccessibility. Therefore, *in vitro* digestion has been used to evaluate the number of substances that become bioaccessible [15,16].

Even though matcha tea may be consumed directly in the powdered form of all leaf parts, there is still little information on its digestibility values and ability to release biologically active substances during digestion. Therefore, a two-step *in vitro* digestion process with pepsin and pancreatin under 37 °C was applied. It aimed to establish the antioxidant activity and contents of individual phenolic acids, flavonoids, xanthine alkaloids, and L-theanine compounds in the native and undigested powdered parts of matcha teas using high performance liquid chromatography (HPLC) with the spectrophotometric and chemiluminescence method. Moreover, the remaining parts (RP) of these compounds in the undigested parts were calculated as well. To determine the contribution of phenolic acids and flavonoids to the antioxidant activity values, the appropriate correlations were evaluated. Scanning electron microscopy (SEM) was applied to monitor the surface of the native and undigested forms of the studied matcha.

## 2. Materials and Methods

### 2.1. Chemicals and Reagents

Pepsin (E.C. 3.4.23.1) with the activity of 0.7 FIG-U/mg and the enzyme mixture of pancreatin with the activities of 350 FIG-U/g of protease, 7500 FIG-U/g of amylase and 6000 FIG-U/g of lipase was purchased from Merck (Darmstadt, Germany). Acetone, HCl, KH_2_PO_4_, Na_2_HPO_4_.12 H_2_O, H_3_PO_4_, CH_3_COOH, and NaOH were provided from Sigma-Aldrich (St. Louis, MI, USA). To determine antioxidant activities, 2,2′ azinobis(3-ethylbenzo-thiazoline-6-sulfonic acid) diammonium salt (ABTS), 2,2-diphenyl-1-picrylhydrazyl (DPPH), 6-hydroxy-2,5,7,8-tetramethylchroman-2-carboxylic acid (trolox), and ascorbic acid (Sigma Aldrich, St. Louis, MO, USA) were used. ACL and ACW kits were supplied by Analytik Jena AG (Jena, Germany). Standards of phenolic acids, flavonoids and alkaloids, and acetonitrile (as a part of a mobile phase) were of HPLC grade (purity > 98.5%) (Sigma-Aldrich, St. Louis, MI, USA).

### 2.2. Matcha Tea Samples

Three different commercial products of matcha tea were purchased in food stores located in Zlín (Czech Republic) in 2021. They included Japan Bio tea (marked as JT) (certified by Organic Crop Improvement Association, originating in Japan) and Allnature and Wolfberry teas (marked as AT and WT, respectively) (both originating in China). All were sold as loose-leaf teas with expiration dates of 2022. They were maintained in the original 0.25–1.00-kg packs out of the sunlight in the air-conditioned laboratory (23 ± 2 °C) for no longer than one month prior to the analysis.

### 2.3. Sample Preparation

Since matcha tea may be consumed in the form of whole leaves and be prepared by cold brewing, this study focuses on the effects of the extraction temperature to simulate cold brewing conditions and human body temperature. Regarding the optimal extraction time under the temperature of 37 °C for phenolic compounds and xanthines from the native and undigested part of matcha infusions, validation parameters were set according to the International Union of Pure and Applied Chemistry to ensure the provision of reliable and interpretable data about the teas. The method was validated in terms of linearity calculated by external calibration, where curves were evaluated from the solution containing phenolic and xanthine analytes, and, after the analysis, the mean values of the four measured areas were correlated with the corresponding concentrations. The correlation coefficients (*r* > 0.9890) indicated that all calibrations maintained linearity within the test ranges. These results suggest that the proposed water extraction method (at 37 °C for 30 min) is appropriate for the quantification [14]. Moreover, the recoveries were calculated in the range of 85 to 108% while RSDs were less than 10%. It demonstrates the extraction method with suitable accuracy and recoveries.

**Figure 1:**
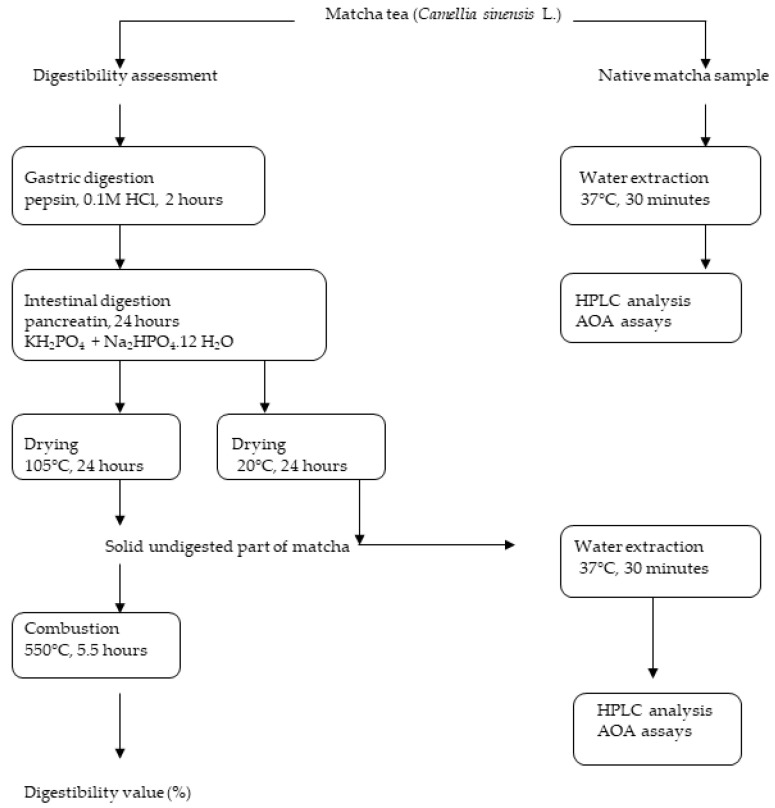
Scheme of *in vitro* digestion procedure. AOA—antioxidant activity assays.

In order to determine the antioxidant activity and content of phenolic acids, flavonoids, caffeine, theobromine and theophylline alkaloids, and L-theanine in the native powders of matcha teas, the samples were first subjected to cold water extraction (Figure 1). A total of 20 mg of the samples was weighed into Eppendorf tubes and mixed with 2 mL of redistilled water. Then, the tubes were placed into a thermo-shaker (TS-100 model, Biosan, Riga, Latvia) at 900 rpm and 37 °C for 30 min. After the tubes were cooled to ambient temperature, the samples were filtered using a nylon syringe filter (13 mm, 0.20 µm) to obtain crude extracts for the HPLC analyses and antioxidant activity assays.

To obtain the undigested powdered residues of matcha tea, the process of digestibility assessment (Section 2.4) was terminated by drying the samples (at 20 °C for 24 h). The solid undigested residue of matcha tea (20 mg) was extracted following the same methodology as described for the native matcha tea samples. All experiments were repeated five times.

### 2.4. In Vitro Gastrointestinal Digestion

Concerning *in vitro* digestibility, the dry matter and ash contents of the samples were determined according to the ISO procedures of 1573 and 1575 [17,18]. The *in vitro* digestion method was conducted following two consecutive steps: gastric and intestinal as is described above [19]. The scheme of the *in-vitro*-simulated digestion procedure is shown in Figure 1. The *in vitro* digestibility of powdered matcha tea was initially assessed with pepsin and then with pancreatin using a Daisy^II^ incubator (Ankom Technology, Macedon, NY, USA). The matcha tea samples were weighed (0.25 g) into F57 type filter bags, washed with acetone, and sealed by the impulse sealer KF-200H (Penta Servis, Holice, Czech Republic). To simulate gastric digestion, an incubator flask was filled with 1.7 L of 0.1 M HCl containing 3 g of pepsin. The sample bags were incubated at 37 °C for 2 h and washed with redistilled water. For the simulation of intestinal digestion, 3.09 g of KH_2_PO_4_ and 32.49 g of Na_2_HPO_4_.12 H_2_O were dissolved in 1.7 L of redistilled water and the pH of the phosphate buffer was set to 7.45 with 0.1 M NaOH. During the second step of digestion, the samples were incubated at 37 °C for 24 h in the phosphate buffer containing of 3 g of pancreatin enzyme. After the incubation, the samples were washed with redistilled water and dried on the filter paper at 105 °C for 24 h. Finally, they were burnt in a muffle furnace at 550 °C for 5.5 h, cooled, and weighed. The *in vitro* digestibility values (%) were calculated according to Equations (1)–(7):

Digestibility (%) = 100 − ((DMR − AR))/(m × DM × OM) × 100(1)

DMR = m3 − m1 × c1(2)

AR = m4 − m1 × c1(3)

DM = (DW × ms)/100(4)

OM = (DW − A)/100(5)

c1 = ms/m1(6)

c2 = mp/m1,(7)

where:

DMR is the weight of the sample without the sack after digestion and drying (g), DM is the dry matter of the sample (%), DW is the dry weight of the sample (g), AAR is the ash weight of the sample with the sack (g), OM is the organic matter content in dry matter of the sample (g), A is the ash content in the sample (g), ms is the weight of the sample for dry matter determination (g), c1 is the correction of the sack weight after incubation (g), c2 is the correction of the sack weight after combustion (g), mp is the weight of ash from the empty correction sack (g), m1 is the weight of an empty bag (g), m2 is the weight of the sample (g), m3 is the weight of a dried bag with the sample after incubation (g), and m4 is the weight of both the sample and the sack after drying and combustion (g).

### 2.5. Determination of Phenolic Acids and Flavonoids Using HPLC

The final extract solutions prepared according to the process described in Section 2.3 were introduced into the liquid chromatograph. Polyphenol compounds were determined according to de Quirós et al. [20] using a Dionex Ultimate 3000 HPLC system equipped with a Dionex Ultimate 3000 Diode Array Detector type DAD-3000RS. The Chromeleon™ 7.2 software was applied (Thermo Fisher Scientific, Waltham, MA, USA) to evaluate the obtained results. Chromatographic separation was performed using a Phenomenex Kinetex C18 column (150 × 4.6 mm; 2.6 µm, Torrance, CA, USA) with the injection volume of 10 µL. The analyses were performed in the gradient mode using redistilled water and acetic acid in the ratio of 99:1 as the mobile phase A (99.8%) and redistilled water:acetonitrile:acetic acid in the ratio of 67:31:1 as the mobile phase B (99.8%). The gradient mode was programmed as follows: a decrease of A to 80% from 0 to 10 min; decrease of A to 60% from 10 to 16 min; decrease of A to 50% from 16 to 20 min; decrease of A to 30% from 20 to 25 min; increase of A to 90% from 25 to 40 min; and constant 90% of A from 40 to 45 min. The flow rate was constant at 1.0 mL/min during the analysis. The separation was performed at 30 °C and chromatograms recorded at 275 nm. The appropriate polyphenol compound was identified according to the retention times of relevant standards. DAD responses were linear for all polyphenol standards within the calibration range of 0.02–120 µg/mL with the correlation coefficients exceeding 0.9990. The results were expressed as µg per g of dry matter. The remaining parts (RP) of all analytes measured in this study were calculated using the equation:

(8) $$\begin{array}{c} RP=\\ \frac{Concentration\;of\;analytes\;in\;undigested\;part\;of\;matcha\;x\;\left(100-digestibility\;value\right)}{Concentration\;of\;analytes\;in\;native\;part\;of\;matcha} \end{array}$$

### 2.6. Determination of Xanthine Alkaloids and L-Theanine Using HPLC

Xanthine alkaloids (caffeine, theobromine, and theophylline) and L-theanine (non-essential amino acid) concentrations in matcha teas were determined according to Boros et al. [12] using HPLC (Thermo Scientific Dionex Ultimate 3000 system with a Dionex Ultimate 3000 Diode Array Detector type DAD-3000RS, Thermo Fisher Scientific, Waltham, MA, USA). The extracts were filtered through a 0.20 µm nylon filter before injection. The injection volume was 10 µL. A Phenomenex Kinetex EVO 18 column (150 mm × 4.6 mm; 5 μm, Torrance, CA, USA) was employed, and the column temperature was set at 25 °C. Mobile phase A was a mixture of redistilled water and 0.05% H_3_PO_4_, and mobile phase B was acetonitrile. The elution gradient started with 100% A with a flow rate of 0.5 mL/min, between the sixth and seventh minute of the analysis the flow rate was altered from 0.5 to 0.8 mL/min. The solution of 100% A with the flow rate of 0.8 mL/min was maintained until the eighteenth minute; it was decreased to 95% A for 15 min followed by a decline to 80% A for 10 min. Subsequently, within one minute, the concentration of the mobile phase A increased from 80 to 100% and was maintained for 9 min. Finally, the flow rate was reduced to 0.5 mL/min within one minute. The detection was performed at the wavelength of 210 nm for L-theanine and 273 nm for theobromine, caffeine, and theophylline alkaloids. The integration and data elaboration were performed using LC Chromeleon™ 7.2 software (Thermo Fisher Scientific, Waltham, MA, USA). Chromatograms were recorded with linear DAD responses within the calibration range of 0–100 µg/mL for L-theanine, 0–600 µg/mL for caffeine, and 0–10 µg/mL for theobromine and theophylline with the correlation coefficients exceeding 0.9985. Each compound was identified using the retention times of relevant standards. The results were expressed as mg or µg per g of dry matter.

### 2.7. Antioxidant Activity Measured Using ABTS and DPPH Radicals

The final extract solutions prepared from the native and undigested powdered matcha samples (Figure 1) were employed in the antioxidant activity assays based on the quenching of the synthetic radicals ABTS^+^ and DPPH. Prior to the analysis, an ABTS stock solution was prepared using 7 mol/L ABTS and 60 mmol/L K_2_S_2_O_8_ in a volume ratio of 1:50 and then incubated at room temperature for 16 h. Thr ABTS working solution was prepared by mixing 2.5 mL of ABTS stock solution and 97.5 mL of acetic buffer (pH 4.3). Two hundred μL of the sample extract were mixed with 24.0 mL of ABTS working solution. Thereafter, the depletion of absorbance was measured spectrophotometrically (Lambda 25, Perkin Elmer, Waltham, MA, USA) at 734 nm after being incubated for 30 min.

Antioxidant activity measurement using DPPH radicals was performed by mixing the extract (450 µL) with 8 mL of DPPH radical solution (0.17 mol/L). The absorbance was monitored at 515 nm after being allowed to rest for 1 h. Trolox in the concentrations of approximately 0 to 60 mg/L was applied as a reference standard in both antioxidant assays [19]. The final results of determination of antioxidant activities were expressed as mg of Trolox equivalent per gram (mg TE/g) of dry matter.

### 2.8. Ferric Antioxidant Power Assay (FRAP)

The analysis was performed according to Esposto et al. [21] using a Lambda 25 spectrophotometer. The extract solutions prepared from the native and undigested powdered form of the matcha samples (Figure 1) were measured. The method is based on the reduction of Fe^3+^-TPZ complexes to their ferrous form at 3.6 pH in the acetic buffer. The reduction was monitored by evaluating the absorption changes at 593 nm. Briefly, 2 mL of FRAP working solution (FeCl_3_:TPZ:acetic buffer = 1:1:10) were mixed with 20–400 µL of the sample, and the absorption was recorded at 37 °C after the 15 min incubation. FRAP values were derived from the subtractions of the absorbance values in the test mixture with the values obtained from the increased concentrations of Fe^3+^. The final results were expressed as mg of Trolox equivalent per gram (mg TE/g) of dry matter.

### 2.9. Antioxidant Activity Determination by Using PCL

The photochemiluminiscence assay (PCL) was performed in accordance with the study by Besco et al. [22] and evaluated using ACL and ACW kits supplied by Analytik Jena AG (Jena, Germany). A photochem device was employed to measure and evaluate the antioxidative potential of lipid (ACL)- and water (ACW)-soluble components. To prepare the Trolox standard solutions, ACL and ACW protocols were applied; in the case of the ascorbic acid standard, only ACW protocol was used. In addition, the results were presented as the integral antioxidant capacity (IAC) quantifying the potential synergistic activity in the particular mixture. The IAC parameter is defined by the sum of the ACW and ACL values of the fractions expressed as mg of TE per gram of dry matter [22,23].

Five hundred µL of reagent 1 (Kit ACL) were placed to the vial containing Trolox (reagent 4, Kit ACL) and shaken for 10–20 s to obtain the stock solution. Then, it was diluted with reagent 1 so that 10 µL of this solution contained 1 nmol Trolox as a standard. Measurements were repeated five times. Similarly, 490 µL of reagent 1 (Kit ACW) and 10 µL H_2_SO_4_ were added to the vial containing ascorbic acid or Trolox (reagent 4, Kit ACW or ACL, respectively) and shaken for 30 s. The resulting stock solution was diluted with reagent 1 to obtain a work solution whose 10 µL contained 1 nmol ascorbic acid or trolox as a calibration standard. The measurements were repeated twice.

The precise quantity of matcha samples (0.5 g) was placed in 5 mL water (ACW) and methanol (ACL) of HPLC grade, sonicated for 15 min, centrifuged (Velocity 13 μ; Dynamica Scientific Ltd., Newport Pargnell, UK) at 12,300 × g for 10 min, and the supernatant was immediately diluted with reagent 1 of ACW or ACL kits (AnalytikJena, Jena, Germany). The results were obtained by converting the molar concentrations to mass using the molar weights for Trolox and ascorbic acid, and they were expressed as mg TE/g (Trolox equivalent) concerning ACW and ACL, respectively; and in mg AAE/g (Ascorbic acid equivalent) for ACW [22]. All results were expressed in terms of dry matter of matcha tea.

### 2.10. Scanning Electron Microscopy (SEM)

The matcha tea powder samples were adhered to a carbon tape on an aluminum target. Powdered forms of the samples subjected to scanning electron microscopy were used in their native forms, after the extraction in water at 37 °C and after the digestibility (as the indigestible fraction). Above that, for comparison, the sample after the water extraction at 70 °C was prepared as well. Unattached powder particles were removed with a stream of compressed dry air. The attached fragments were examined using SEM (Phenom Pro model, Phenom-World BV, Thermo Fisher Scientific, Eindhoven, The Netherlands). The samples were analyzed at an acceleration voltage of 10 kV in a backscattered electron mode with the magnification levels of 1500×, 3500×, and 10,000×. Measurements performed on the samples without prior metallization were completed using a holder allowing the reduction of charges on polymeric materials.

### 2.11. Statistical Analysis

All analyses were repeated five times, and the results were expressed as mean ± standard deviation (SD) in dry matter (the mean of five measurements). The analysis of variance (ANOVA) was applied. Subsequently, Tukey´s test was employed to identify the differences among the means. The significance level of probability was set to 5%. The correlations were defined using Pearson´s correlation coefficient (*r*).

## 3. Results and Discussion

### 3.1. Dry Matter and Ash Contents and In Vitro Digestibility Values

Dry matter, ash contents, and *in vitro* digestibility values of matcha teas are shown in Table 1. Dry matter contents varied from 95.4 to 96.9%. According to Reg. No. 330 [24], the moisture content for green tea should not exceed 10%. Regarding ash content, ISO 11287 [25] determines the minimum and maximum levels of ash as 4 and 8%, respectively. The ash content in this study varied between 4.64 and 4.99%. Topuz et al. [9] stated a positive relationship between the quality of the green tea and the ash amount and proposed that for a good quality tea the ash content should comprise less than 5.54%. Even though data evaluating digestibility values of matcha teas are scarce, these results are comparable to the study provided by Koláčková et al. [19], where digestibility values of matcha teas ranged from 59.4 to 69.7%. It is known that low digestibility values positively correlate with a high content of a dietary fiber. Moreover, high dietary fiber content decreases protein and starch digestibility. It has also been reported that protein digestibility values are significantly reduced by tannins [26].

### 3.2. Effect of In Vitro Digestibility on Phenolic Acids and Flavonoids of Matcha Tea

As matcha tea contains all leaf parts, it is important to monitor the concentrations of individual substances in both the native and undigested portion of matcha tea (Table 2). The comparison of the results with other studies is limited due to scarce data provided in the same conditions of the matcha extracts’ preparation to study digestibility as well as to obtain an indigestible portion of leaves for further analysis. Table 2 provides the values of the individual phenolic acids and flavonoids, total phenolic acids, flavonoids, and total individual phenolic values determined in the native and undigested portions of matcha tea.

Regarding phenolic acid compounds in the native matcha tea samples, chlorogenic acid (2090–2460 µg/g) was identified as the most significant phenolic acid followed by ellagic (181–236 µg/g), sinapic (63.7–85.1 µg/g), gallic (37.9–91.1 µg/g), cinnamic (23.6–67.4 µg/g), and protocatechuic acid (24.9–54.1 µg/g). Chlorogenic acid found in the AT matcha was the highest (2460 µg/g) phenolic acid determined with cold water extraction at 37 °C. Gallic and chlorogenic acids are generally monitored as the main phenolic acids in green teas when the hot water steeping process of about 70–80 °C was applied [3,27].

Matcha is grown in 90% shade. As a result, concentrations of flavonoids available from matcha are expected to differ. The 30 min water extraction simulating the temperature of the human body (37 °C must be considered as well. As shown in Table 2, the highest flavonoid contents of native matcha teas were established in the order of epigallocatechin-3-gallate (EGCG), epicatechin (EC), epigallocatechin (EGC), and epicatechin-3-gallate (ECG), and their amounts changed in the ranges of 7610–16,900 µg/g, 5140–14,500 µg/g, 4210–5390 µg/g, and 1520–1790 µg/g, respectively. The concentration range of rutin exceeded that of catechin, 303–479 µg/g and 10.2–23.1 µg/g, respectively. Catechin is known to be susceptible to degradation due to several factors, including pH, temperature, oxygen, and metal ions. [8]. The epicatechin concentrations in the green teas were measured in the range of 4.29–8.61 mg/g within the water extraction performed at 80 °C for 20 min [3]. Moreover, the epigallocatechin and catechin contents of young shoot tea leaves grown in the shade area are lower if covered. It has been reported that the levels of epicatechin increased from the bud to the second leaf [28,29]. In addition, catechin has been reported to be highly influenced by the various storage conditions [8]. Epigallocatechin-3-gallate (EGCG) found in the WT sample was the most abundant (16,900 µg/g) flavonoid in the water-extraction procedure (Table 2). Catechin, EC, ECG, EGC, and EGCG were indicated as predominant flavonoids and phenolic acids found in green tea as well [1,7].

The concentrations of individual polyphenols established after *in vitro* digestion in the undigested parts of matcha teas were used to calculate the remaining parts of the compounds (RP, %). The results are displayed in Table 2. It can be seen that ferulic, ellagic, and caffeic acid were the least-released phenolic acids, and their RP values reached 84, 80 and 76%, respectively. Protocatechuic, 4-hydroxybenzoic, neochlorogenic acid, and protocatechuic acid ethyl ester were potentially more accessible compounds in the human digestive tract. Due to the deficiency of studies related to bioaccessibility of individual phenolic compounds analyzed in matcha tea either in the native or undigested part, it is very difficult to compare the results obtained in this study with other findings. Focusing on cereals, Sęczyk et al. [30] found the highest and lowest bioaccessibility values of *p*-coumaric (up to 93%) and gallic acid (23.7%), respectively. Drawbridge et al. [31] suggested that vanillic acid might be less susceptible to degradation during digestion than *p*-coumaric and sinapic acid. It can be seen that only 9% of unreleased vanillic acid remained in the undigested part of matcha tea samples (Table 2). Theoretically, vanillic acid may be absorbed by the human digestive tract by more than 90%. However, its bioaccessibility from barley grains was about 85% [31].

The least digestible/released flavonoids were rutin and quercetin; on the other hand, epigallocatechin (EGC) was the highest digestible flavonoid followed by epigallocatechin-3-gallate (EGCG), epicatechin (EC), and catechin (C) (Table 2). They can be used in the digestive tract, unless they degrade. It should be emphasized that *o*-coumaric acid and epicatechin-3-gallate (ECG) were not detected in the undigested parts of matcha. It can only be hypothesized that they may be completely released from the tea-leaf matrix and degraded. This assumption has been verified only by an *ex vivo* study by Dai et al. [32], who claimed the low stability of EGCG in the small intestine and suggested its delivery through nanoparticles. It is generally known that the bioaccessibility of polyphenols and other biologically active compounds is significant. Considering their biological importance, the interactions of substances with polyphenols in the food matrix are very complex. During digestion, many biochemical (presence of enzymes, electrolytes) and physicochemical (pH, temperature) factors influence the stability of dietary polyphenol compounds and affect their release from the food matrix [30]. It has been reported that the bioaccessibility of catechins from green tea was positively affected by adding β-lactoglobulin due to the elimination of the protein-catechin interactions [33].

Recently, cold water (25–40 °C) steeping is more popular in green tea preparation. Not only the temperature and amount of water, but matcha tea quantity also has critical importance. Moreover, there are further factors to be considered, such as the time of infusion or particle size. In addition, the phytochemical composition of tea is affected by the cultivation conditions, including agricultural practices, location, cultivar, the life cycle of tea leaf, and shelf-life during storage [34,35]. However, the appropriate dose levels of phenolic compounds required for the physiological activity of the human body cannot be identified from the reported findings. Green teas have been studied extensively for their flavonoid and phenolic acid contents and antioxidant activity [2,6,34]. Unfortunately, there is only little data on the undigested residues of matcha tea. This study shows that both flavonoids and phenolic acids are included in the undigested residue of matcha and could pass into the colon. Further investigation of the undigested and released polyphenolic compounds and their stability after digestion is necessary.

### 3.3. The Effect of In Vitro Digestibility on Xanthine Alkaloids and L-Theanine

The concentrations of caffeine, theobromine and theophylline alkaloids, and L-theanine in the native and undigested parts of matcha teas are presented in Table 3. It has been observed that caffeine was presented in the highest concentration in the range of 14.1–16.1 mg/g while theophylline exhibited the lowest concentration in the range of 8.06–19.4 µg/g in the matcha tea samples. It has been reported that the caffeine concentrations transferred to matcha ice teas were significantly influenced by the water temperature. High temperatures responded to a higher yield of caffeine. In addition, the age of green leaves is also a very important factor, as young leaves contain higher caffeine contents when compared to old leaves [1,28,34]. For instance, the levels of caffeine in green teas ranged between 26.7–38.1 mg/g at the extraction conditions of 80 °C and 20 min [3]. L-theanine identified as the major free non-essential amino acid in green tea is fundamentally responsible for the flavor [5]. It has been observed that green tea contained high amounts of L-theanine (6.56 mg/g), whereas the ratio of caffeine/L-theanine was remarkably low (2.79) [12]. Regarding the native matcha tea samples, L-theanine contents varied between 4.22–9.85 mg/g, and the caffeine/L-theanine ratio ranged between 1.63–3.43. The higher value of L-theanine in Japan Bio tea sample (9.85 mg/g) could be attributed to the shading technique which allowed the synthesis a high amount of L-theanine as well as caffeine [2,10,13]. Although the interaction between caffeine and L-theanine has not been defined in detail yet, it can be stated that samples with a lower caffeine/L-theanine ratio have a less pronounced stimulating effect [12]. The high content of L-theanine and caffeine and the low content of catechin result in the umami taste of tea [2]. Friedman et al. [36] recorded the theobromine concentrations in green tea leaves in the range of 0.04–1.90 mg/g if the leaves were treated with boiling water for 10 min. The levels of theobromine monitored in this study were comparable with that research. In addition, Friedman et al. [36] observed that the average content of theobromine was approximately 1/10 of the caffeine content which have not been confirmed in this study. Similarly, Azevedo et al. [14] reported the content of theobromine in green teas as being between 3.95–8.39 mg/g when leaves were extracted at 80 °C for 3 min. There is limited data dealing with the extraction of theobromine by cold brewing. The variability of the caffeine levels among the samples was significantly smaller than that of the theophylline levels (8.06–19.4 µg/g). Theophylline, which is present in trace amounts in *C. sinensis* teas, has a similar physiological effect to caffeine [37]. Extremely high amounts of theophylline (1.99 mg/g) were established in green tea of Sullulah originated in India in the study by Sharma et al. [38].

The results indicated that small amounts of caffeine and trace concentrations of L-theanine were detected in the undigested parts of matcha tea samples. The maximum value of 13% caffeine remained in the undigested part of matcha tea. Theobromine and theophylline were not detected in the undigested parts of matcha; therefore, they may have been released during digestion and absorbed by the digestive tract. Owing to the limited data devoted to the digestibility and bioaccessibility of alkaloids from matcha tea, it is complicated to compare the results obtained in this study.

### 3.4. Results of Antioxidant Activity Measurements

The effect of *in vitro* digestibility on the antioxidant activity values was provided using four independent assays. Table 4 depicts the results showing that matcha tea infusions have a significant antioxidant potential. The highest values were observed for both the native and indigestible parts of the Wolfberry tea samples determined by the quenching of ABTS and DPPH radicals as well as the reduction of Fe^3+^-TPZ complexes.

Concerning the PCL method, the ACW values of the water-soluble antioxidants were between 194–209 and 1.10–1.33 mg AAE/g for the native and indigestible parts of matcha, respectively. Similarly, the ACL values of lipid-soluble substances ranged between 162–179 and 6.25–7.12 mg TE/g, respectively. To evaluate the antioxidant capacity as an IAC parameter, the activity of the water-soluble compounds (ACW) was measured and expressed not only in AAE/g but also in mg TE/g. The results obtained by the chemiluminescence assay emphasize that the water-soluble biological compounds of matcha tea (ACW values) show higher values of antioxidant activity only in the case of the native matcha samples. Regarding the undigested residue of matcha, higher values of antioxidant activity were established for the lipid-soluble compounds (ACL values). The total IAC values of the native matcha tea reached 467 mg TE/g, in the case of the indigestible part 12.7 mg TE/g. These results show that biologically active substances may be present in the form of indigestible matcha and thus have only inconsiderable antioxidant potential. Evaluating all methods determining antioxidant activity, the values were reduced by more than 94% after the simulation of the *in vitro* digestion process.

Antioxidant properties are influenced by many factors, such as a brewing time, brewing temperature, and water yield during the extraction of tea compounds, and cannot be described precisely by only one method. It has been confirmed that using lower extraction temperatures in combination with longer extraction times may enhance antioxidant activity by protecting the specific phenolic fractions of the plant material [2,27,34]. Due to the lack of data providing information about the effects of *in vitro* gastrointestinal digestion on phenolics, xanthine alkaloids and antioxidant activity of matcha tea samples, it is difficult to compare the obtained values with already published results. Since antioxidants are either water or fat soluble, the research in the food industry has experienced a growing interest in the PCL antioxidant activity assay. The results obtained from the PCL express IAC value since the radicals are eliminated by a mixture of antioxidants. Some of them has their own biological effect either in the hydrophilic or lipophilic body compartments [22]. Generally, higher ACW values in fruits and teas are usually attributed to high contents of ascorbic acid, catechins and other flavonoids, and amino acids, while tocopherols, tocotrienols, carotenoids, and the effect of *in vitro* digestibility are considered to be sources of antioxidant activity in the lipid-soluble fractions (ACL). Due to the fact that not only a higher content of polyphenolic acids and flavonoids, but also vitamin C and chlorophyll were measured in matcha teas [19], it may be assumed that these biologically active substances also contribute to the ACW and ACL values.

All antioxidant activity assays used in this study adopt their own chemical reaction mechanism [21]. Phenolics, especially catechins, in green teas have been known to have antioxidant properties [1,2,8,21]. However, there is little information about the contribution of individual phenolics to their overall antioxidant activity in the undigested part of matcha tea after the *in-vitro*-simulated digestion method. Each phenolic compound performs a different activity depending on the number of aromatic and hydroxyl groups and its distribution in their molecules and is influenced by a chemical form to which they are bound. The interaction type depends greatly on the model system and the conditions of the analysis (e.g., *in vivo* or *in vitro* digestion, bioavailability, temperature, extract solution). Certain polyphenols in the tested food matrices would be consumed as glycosides, and further factors, such as the enzyme activity, digestive factors and other foods, may be present at the same time to influence the interactions [19,39,40]. Table 5 and Table 6 shows the correlations between the contents of individual phenolic acids and flavonoids and antioxidant activity. They were calculated to identify the major contributors to the antioxidant activity values of the phenolic fractions in the native and undigested parts of matcha tea.

Concerning the ABTS, DPPH, and FRAP methods, the main contributors to the antioxidant activity in the native form of matcha tea appear to be 4-hydroxybenzoic acid > caffeic acid > sinapic acid > *o*-coumaric acid > gallic acid > catechin > epigallocatechin-3-gallate > rutin > syringic acid. In terms of the undigested part of matcha samples, it may be emphasized that the main phenolic contributors to the antioxidant activity are in the following order: sinapic acid > cinnamic acid > protocatechuic acid ethyl ester > neochlorogenic acid > epigallocatechin-3-gallate > 4-hydroxybenzoic acid > caffeic acid > epigallocatechin > catechin > syringic acid. These results may be compared with the study by Koláčková et al. [19] stating ellagic, protocatechuic, chlorogenic, and *p*-hydroxy-benzoic acids as the main contributors to the antioxidant activity values in the native matcha tea samples. Phenolic concentrations depend on the growing conditions, such as the temperature, light, and further climatic factors; the amount of leaves used per water amount; the extraction time; and water temperature.

Regarding the water-soluble antioxidants (ACW) of the native samples measured using PCL kits, the main contributors to the antioxidant activity values include gallic, syringic and cinnamic acids, epigallocatechin, sinapic, caffeic, 4-hydroxybenzoic, and neochlorogenic acids. Epigallocatechin, neochlorogenic, ellagic, cinnamic, vanillic, syringic acids, epigallocatechin-3-gallate, and quercetin seem to be strong antioxidants in the lipophilic fraction (ACL) of the native samples. In terms of the undigested part of matcha, the main contributors to the ACW values are cinnamic and neochlorogenic acids, epigallocatechin-3-gallate, epigallocatechin, protocatechuic acid ethyl ester, and syringic and 4-hydroxybenzoic acids. Similarly, concerning the hydrophobic antioxidants (ACL) of the undigested parts of matcha, the order of significant providers of the antioxidant activity is as follows: epicatechin > quercetin > ellagic acid > vanillic acid > syringic acid > epigallocatechin > epigallocatechin-3-gallate. Although theoretical correlations indicate the presence of polyphenolic substances that might contribute to the antioxidant activity values of undigested tea leaves, we must consider that the residual antioxidant activity value of the undigested portion is very low (below 6%) (Table 4).

### 3.5. SEM

**Figure 2:**
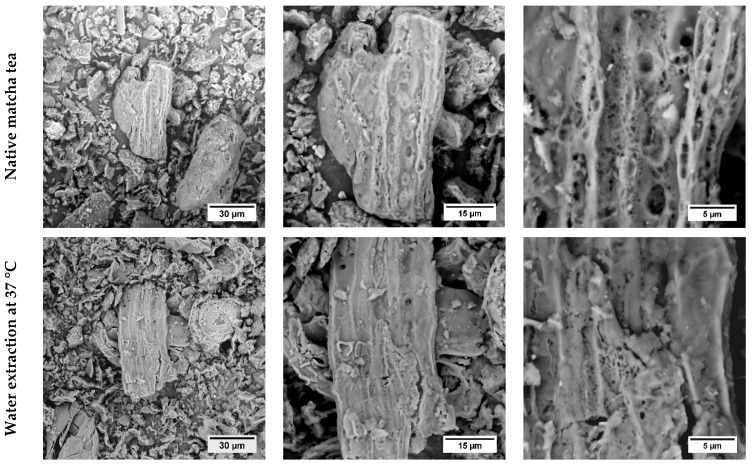

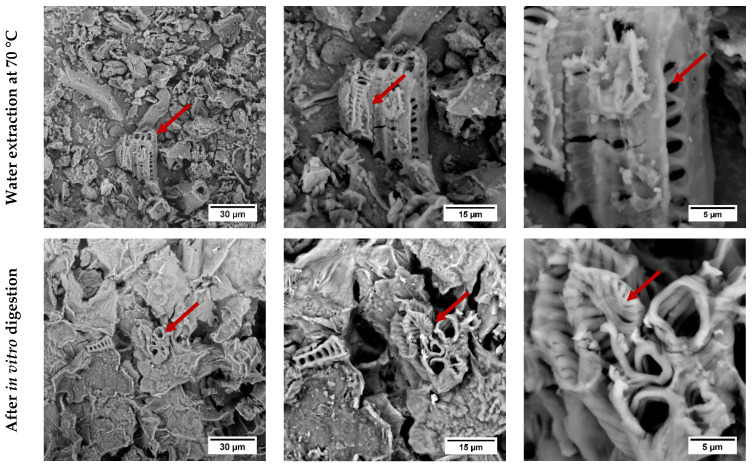
SEM images of the native matcha tea powder (the first row from the top), water extract at 37 °C (the second row from the top), water extract at 70 °C (the third row from the top), and matcha tea after in vitro digestion (the fourth row from the top) obtained at the magnification levels of 1500× (the first column), 3500× (the second column), and 10,000× (the third column) for each sample.

SEM images of the native matcha tea powder (the first row from the top), water extract at 37 °C (the second row from the top), water extract at 70 °C (the third row from the top), and matcha tea after *in vitro* digestion (the fourth row from the top) obtained at the magnification levels of 1500× (the first column), 3500× (the second column), and 10,000× (the third column) are shown in Figure 2.

As can be seen, there was no significant degradation observed in the native water extract of matcha tea at 37 °C. The powder matcha sample was subjected to a further analysis at the temperature of 70 °C to observe if any changes occurred in the surface structure. Similar decomposition processes leading to the formation of specific structures indicated by red arrows were monitored for the undigested part of matcha powder and also the powder after the application of 70 °C. The effect of the particle size on the extraction processes and *in vitro* digestion have been observed. Even though studies examining the effect of matcha tea particle sizes on the digestibility and bioaccessibility of polyphenol compounds is scarce, Shu et al. [41] stated that the particle sizes of green tea leaves (D50 values were 564, 75, and 35 µm) significantly influenced the bioaccessibility values of polyphenol compounds (especially catechin flavonoid) after *in vitro* digestion. The bioaccessibility of polyphenol compounds significantly increased with the reduction of the particle size. Furthermore, cellulose and hemicellulose degradation during grinding may have contributed as well. Hopefully, similar assumptions can be made for matcha tea in future research.

## 4. Conclusions

This study investigates the effect of *in vitro* digestion on phenolic acids, flavonoids, xanthine alkaloids and L-theanine content, and antioxidant values of commercial matcha tea samples. It also evaluates the correlations between phenolics and antioxidant activity values. Moreover, it examines the effect of *in vitro* digestibility on the structures of matcha tea powders using SEM. The *in vitro* digestibility values of the analyzed matcha tea samples were 65.8, 64.9, and 61.2%, respectively. Considering flavonoids in the native matcha samples, 30 min water extraction at 37 °C showed the order of the highest contents of flavonoids as follows: epigallocatechin-3-gallate (EGCG) > epicatechin (EC) > epigallocatechin (EGC) > epicatechin-3-gallate (ECG). It should be emphasized that the concentration range of rutin exceeded that of catechin. Chlorogenic acid was determined as the prevalent phenolic acid followed by ellagic, sinapic, gallic, cinnamic, and protocatechuic acids. Concerning the undigested parts of the matcha samples, the highest concentrations of rutin, epicatechin, chlorogenic, and ellagic acids were established. The amount of the compound’s remaining parts (RP) after the application of *in vitro* digestion was calculated as well. The highest RP values were monitored for rutin (78–82%) and quercetin (61–78%). It can be stated that ferulic, ellagic, and caffeic acid were the least digestible/released phenolic acids, while epigallocatechin, epigallocatechin-3-gallate, epicatechin, catechin, protocatechuic, 4-hydroxybenzoic and neochlorogenic acids, and protocatechuic acid ethyl ester appeared to be easily released and might be potentially more accessible for absorption by the human intestine. Discussing the results of xanthine alkaloids and L-theanine, the native extract of matcha tea provided the highest values of caffeine, while theophylline was established in the lowest concentration. Focusing on the undigested parts of matcha, trace concentrations of L-theanine and reduced contents of caffeine were determined. It has been found that phenolics, caffeine, and L-theanine may be present in the form of indigestible matcha and may have only inconsiderable antioxidant potential. Antioxidant activity values in the undigested parts of the samples were smaller by more than 94% after the application of *in vitro* digestion process. The results of the correlation analysis of the native matcha samples showed the main contributors to the antioxidant activity (measured using ABTS, DPPH and FRAP) in the order: 4-hydroxybenzoic acid > caffeic acid > sinapic acid > *o*-coumaric acid > gallic acid > catechin > epigallocatechin-3-gallate. The main contributors to the antioxidant activity in the undigested part of matcha were sinapic acid > cinnamic acid > protocatechuic acid ethyl ester > neochlorogenic acid > epigallocatechin-3-gallate > 4-hydroxybenzoic acid > caffeic acid > epigallocatechin > catechin > syringic acid. Furthermore, the photochemiluminiscence assay (PCL) showed that strong, water-soluble antioxidants of native matcha were gallic, syringic, cinnamic acids, epigallocatechin, sinapic, caffeic, 4-hydroxybenzoic, and neochlorogenic acids, while epigallocatechin, neochlorogenic, ellagic, cinnamic, vanillic, syringic acids, epigallocatechin-3-gallate, and quercetin were strong antioxidants in the lipophilic fraction. Regarding the undigested parts of matcha, cinnamic and neochlorogenic acids, epigallocatechin-3-gallate, epigallocatechin, protocatechuic acid ethyl ester were established as the main contributors to ACW values. Concerning hydrophobic antioxidants (ACL), significant donators to the antioxidant activity were established in the order of epicatechin > quercetin > ellagic acid > vanillic acid > syringic acid > epigallocatechin > epigallocatechin-3-gallate. It must be considered that the residual antioxidant activity of the undigested residue was less than 6%. The disruption of matcha tea powder during *in vitro* digestion was monitored using SEM. It has shown that some polyphenol compounds might be retained in the matcha’s undigested residue and enter the large intestine. This hypothesis requires further future investigation.

## Figures and Tables

**Table 1:** Dry matter and ash contents and *in vitro* digestibility values of matcha teas.

Values	Dry Matter (%)	Ash ^1^ (%)	Digestibility (%)
JT	96.9 ± 0.3 ^a^	4.99 ± 0.05 ^a^	65.8 ± 0.3 ^a^
AT	96.2 ± 0.3 ^a^	4.85 ± 0.05 ^b^	64.9 ± 0.5 ^b^
WT	95.4 ± 0.4 ^b^	4.64 ± 0.05 ^c^	61.2 ± 0.4 ^c^

All results are presented as means ± SD, *n* = 5 (the mean of five measurements). Means within a column with at least one identical small superscript do not differ significantly (*p ≥* 0.05). ^1^ Presented in dry matter basis. JT—Japan bio tea, AT—Allnature tea, WT—Wolfberry tea. Dry matter was assessed according to ISO procedures of 1573 [17].

**Table 2:** Phenolic acid and flavonoid compounds determined in native and undigested parts of matcha teas.

Analytes	Native JT	Native AT	Native WT	Undigested JT	Undigested AT	Undigested WT	RP (%)
Phenolic acids (µg/g)							
GA	60.4 ± 2.0 ^a^	37.9 ± 0.3 ^b^	91.1 ± 1.0 ^c^	8.11 ± 0.20 ^A^	19.7 ± 0.5 ^B^	19.5 ± 0.5 ^B^	5–18
PA	43.2 ± 2.0 ^a^	54.1 ± 1.0 ^b^	24.9 ± 1.0 ^c^	0.25 ± 0.02 ^A^	0.31 ± 0.02 ^B^	0.12 ± 0.01 ^C^	<1
VA	2.58 ± 0.10 ^a^	1.47 ± 0.05 ^b^	1.31 ± 0.03 ^c^	0.51 ± 0.02 ^A^	0.36 ± 0.02 ^B^	0.22 ± 0.01 ^C^	7–9
SA	4.34 ± 0.06 ^a^	1.98 ± 0.04 ^b^	4.36 ± 0.05 ^a^	1.38 ± 0.05 ^A^	1.24 ± 0.02 ^B^	1.29 ± 0.02 ^C^	11–22
PAEE	9.22 ± 0.30 ^a^	8.80 ± 0.12 ^b^	4.26 ± 0.13 ^c^	0.06 ± 0.01 ^A^	0.05 ± 0.01 ^A^	0.10 ± 0.01 ^B^	<1
EA	236 ± 5 ^a^	184 ± 5 ^b^	181 ± 4 ^b^	452 ± 10 ^A^	401 ± 7 ^B^	359 ± 7 ^C^	66–80
4-hBA	12.9 ± 0.1 ^a^	13.3 ± 0.2 ^b^	14.6 ± 0.2 ^c^	0.14 ± 0.01 ^A^	0.14 ± 0.01 ^A^	0.17 ± 0.01 ^B^	<1
*trans-p*-CA	9.11 ± 0.04 ^a^	13.9 ± 0.3 ^b^	13.3 ± 0.2 ^c^	2.57 ± 0.10 ^A^	5.35 ± 0.20 ^B^	4.23 ± 0.10 ^C^	10–13
CA	2.31 ± 0.10 ^a^	2.46 ± 0.12 ^b^	4.09 ± 0.20 ^c^	5.12 ± 0.10 ^A^	5.26 ± 0.03 ^B^	7.17 ± 0.10 ^C^	68–76
FA	12.7 ± 0.2 ^a^	14.4 ± 0.2 ^b^	12.6 ± 0.3 ^a^	25.6 ± 0.5 ^A^	32.9 ± 0.6 ^B^	27.2 ± 0.2 ^C^	69–84
SiA	63.7 ± 1.1 ^b^	65.4 ± 2.0 ^b^	85.1 ± 2.5 ^a^	24.8 ± 0.2 ^B^	21.0 ± 0.1 ^C^	26.7 ± 0.3 ^A^	11–13
ChA	2310 ± 50 ^a^	2460 ± 40 ^b^	2090 ± 40 ^c^	512 ± 10 ^A^	526 ± 7 ^B^	517 ± 10 ^A^	7–10
NeoChA	27.0 ± 0.5 ^a^	12.5 ± 0.3 ^b^	14.4 ± 0.2 ^c^	0.60 ± 0.02 ^A^	0.12 ± 0.01 ^B^	0.52 ± 0.05 ^C^	1–3
*o*-CA	6.33 ± 0.20 ^a^	6.80 ± 0.03 ^b^	7.00 ± 0.10 ^b^	ND	ND	ND	–
CiA	67.4 ± 2.0 ^a^	23.6 ± 1.0 ^b^	60.7 ± 1.2 ^c^	26.5 ± 1.0 ^A^	12.5 ± 0.4 ^B^	35.2 ± 0.7 ^C^	14–23
T-PAs	2870 ± 50 ^a^	2900 ± 40 ^a^	2610 ± 40 ^b^	1060 ± 20 ^A^	1030 ± 10 ^B^	1000 ± 10 ^C^	12–15
Flavonoids (µg/g)							
EGC	5390 ± 50 ^a^	4210 ± 40 ^b^	4850 ± 40 ^c^	15.0 ± 0.4 ^A^	12.6 ± 0.2 ^B^	14.1 ± 0.1 ^C^	<1
C	10.2 ± 0.5 ^a^	23.1 ± 1.0 ^b^	17.8 ± 0.5 ^c^	1.01 ± 0.02 ^A^	1.30 ± 0.05 ^B^	1.95 ± 0.05 ^C^	2–4
EC	8940 ± 40 ^a^	14500 ± 100 ^b^	5140 ± 30 ^c^	650 ± 10 ^A^	320 ± 10 ^B^	160 ± 10 ^C^	1–3
EGCG	15300 ± 100 ^a^	7610 ± 50 ^b^	16900 ± 100 ^c^	51.6 ± 0.4 ^A^	28.9 ± 0.6 ^B^	44.8 ± 0.3 ^C^	< 1
ECG	1640 ± 40 ^a^	1790 ± 40 ^b^	1520 ± 40 ^c^	ND	ND	ND	–
R	303 ± 10 ^a^	479 ± 20 ^b^	356 ± 15 ^c^	725 ± 10 ^A^	1060 ± 30 ^B^	746 ± 10 ^C^	78–82
Q	11.0 ± 0.3 ^a^	9.22 ± 0.12 ^b^	8.02 ± 0.20 ^c^	25.0 ± 0.5 ^A^	16.1 ± 0.3 ^B^	15.2 ± 0.3 ^C^	61–78
T-FLs	31600 ± 150 ^a^	28600 ± 150 ^b^	28800 ± 140 ^c^	1470 ± 10 ^A^	1440 ± 30 ^B^	982 ± 10 ^C^	19–41
Total Phe	34500 ± 150 ^a^	31500 ± 150 ^b^	31400 ± 100 ^c^	2530 ± 20 ^A^	2470 ± 30 ^B^	1980 ± 20 ^C^	16–38

All results are presented in dry matter as means ± SD, *n* = 5 (the mean of five measurements). Means within a line with at least one identical small superscript (native matcha) do not differ significantly (*p* ≥ 0.05), means within a line with at least one identical capitalized superscript (undigested parts of matcha) do not differ significantly (*p* ≥ 0.05). JT—Japan bio tea, AT—Allnature tea, WT—Wolfberry tea, RP—Remaining part of compound, GA—gallic acid, PA—protocatechuic acid, VA—vanillic acid, SA – syringic acid, PAEE—protocatechuic acid ethyl ester, EA—ellagic acid, 4-hBA—4-hydroxy benzoic acid, *trans-p*-CA—*trans-p*-coumaric acid, CA—caffeic acid, FA—ferulic acid, SiA—sinapic acid, ChA—chlorogenic acid, NeoChA—neochlorogenic acid, *o*-CA—*o*-coumaric acid, CiA—cinnamic acid, EGC—epigallocatechin, C—catechin, EC—epicatechin, EGCG—epigallocatechin-3-gallate, ECG—epicatechin-3-gallate, R—rutin, Q—quercetin, T-PAs—total phenolic acids, T-FLs—total flavonoids, Total Phe—total individual phenolics. LOQ: quercetin 0.02 µg/g, epicatechin-3-gallate 1.00 µg/g.

**Table 3:** Xanthine alkaloid and L-theanine compounds determined in native and undigested part of matcha teas.

Compounds	Native JT	Native AT	Native WT	Undigested JT	Undigested AT	Undigested WT	RP (%)
(mg/g)							
Caffeine	16.1 ± 0.3 ^a^	14.1 ± 0.2 ^c^	15.3 ± 0.1 ^b^	5.26 ± 0.12 ^A^	3.66 ± 0.10 ^B^	4.99 ± 0.15 ^C^	9–13
L-theanine	9.85 ± 0.10 ^a^	4.22 ± 0.05 ^c^	4.46 ± 0.10 ^b^	0.15 ± 0.01 ^A^	0.09 ± 0.01 ^B^	ND	<1
Theobromine	0.14 ± 0.02 ^c^	0.27 ± 0.02 ^a^	0.23 ± 0.02 ^b^	ND	ND	ND	-
(µg/g)							
Theophylline	8.06 ± 0.15 ^c^	19.4 ± 0.2 ^a^	13.0 ± 0.2 ^b^	ND	ND	ND	-
The ratio of Caffeine/ L-theanine	1.63	3.34	3.43				

All results are presented in dry matter as means ± SD, *n* = 5 (the mean of five measurements). Means within a line with at least one identical small superscript (in case of native matcha) do not differ significantly (*p* ≥ 0.05), means within a line with at least one identical capitalized superscript (in case of undigested part of matcha) do not differ significantly (*p* ≥ 0.05). JT—Japan bio tea, AT—Allnature tea, WT—Wolfberry tea, RP—Remaining part of compound. LOQ: L-theanine and theobromine 0.05 µg/g, theophylline 0.02 µg/g.

**Table 4:** Antioxidant activity (AOA) values determined in native and undigested part of matcha teas.

Antioxidant Activity	Native JT	Native AT	Native WT	Undigested JT	Undigested AT	Undigested WT
ABTS (mg TE/g)	187 ± 10 ^a^	249 ± 5 ^b^	305 ± 12 ^c^	10.5 ± 0.2 ^A^ − 94%	3.82 ± 0.15 ^B^ − 98%	13.4 ± 0.3 ^C^ − 96%
DPPH (mg TE/g)	121 ± 4 ^a^	134 ± 5 ^b^	176 ± 10 ^c^	7.00 ± 0.20 ^A^ − 94%	3.14 ± 0.05 ^B^ − 98%	8.89 ± 0.20 ^C^ − 95%
FRAP (mg TE/g)	102 ± 3 ^a^	125 ± 4 ^b^	162 ± 6 ^c^	5.16 ± 0.10 ^A^ − 95%	4.33 ± 0.10 ^B^ − 97%	6.12 ± 0.20 ^C^ − 96%
ACW (mg AAE/g)	202 ± 7 ^a^	194 ± 6 ^b^	209 ± 7 ^c^	1.26 ± 0.03 ^A^ − 99%	1.10 ± 0.01 ^B^ − 99%	1.33 ± 0.03 ^C^ − 99%
ACW (mg TE/g)	288 ± 9 ^a^	276 ± 7 ^b^	293 ± 10 ^a^	5.55 ± 0.15 ^A^ − 98%	4.97 ± 0.12 ^B^ − 98%	5.67 ± 0.14 ^A^ − 98%
ACL (mg TE/g)	179 ± 5 ^a^	162 ± 7 ^b^	169 ± 6 ^c^	7.12 ± 0.20 ^A^ − 96%	6.47 ± 0.20 ^B^ − 96%	6.25 ± 0.30 ^C^ − 96%
IAC (mg TE/g)	467 ± 10 ^a^	438 ± 10 ^b^	462 ± 12 ^c^	12.7 ± 0.3 ^A^ − 97%	11.4 ± 0.2 ^B^ − 97%	11.9 ± 0.3 ^C^ − 97%

All results are presented as means in dry matter ± SD, *n* = 5 (the mean of five measurements). Means within a line with at least one identical small superscript (in case of native matcha) do not differ significantly (*p* ≥ 0.05), means within a line with at least one identical capitalized superscript (in case of undigested part of matcha) do not differ significantly (*p* ≥ 0.05). The decrease in values of antioxidant activity after gastrointestinal treatment is presented in percentage (calculation example: 100−((10.5/187) × 100)). JT—Japan bio tea, AT—Allnature tea, WT—Wolfberry tea, ABTS—antioxidant activity measured using 2,2´-azinobis(3-ethylbenzo-thiazoline-6-sulfonic-acid) radical, DPPH—antioxidant activity measured using 2,2-diphenyl-1-picrylhydrazyl radical, FRAP—antioxidant activity measured using 2,4,6-tripyridyl-s-triazine, ACW—antioxidant capacity of water-soluble compounds, ACL—antioxidant capacity of lipid-soluble compounds, TE—Trolox equivalent, AAE—ascorbic acid equivalent, IAC—integral antioxidant capacity.

**Table 5:** The relation of several evaluation parameters of native matcha tea.

	Antioxidant Activity Values						
*r*	ABTS	DPPH	FRAP	ACW	^1^ ACW	ACL	IAC
Phenolics							
GA	0.5505	0.7882	0.6789	0.9919	0.9486	0.3272	0.7149
PA	−0.5968	−0.8217	−0.7193	−0.9832	−0.9292	−0.2733	−0.6742
VA	−0.9293	−0.7628	−0.8569	−0.0774	0.1172	0.8587	0.5393
SA	0.0220	0.2982	0.1407	0.8880	0.9603	0.8066	0.9857
PAEE	−0.8886	−0.9885	−0.9513	−0.8030	−0.6723	0.1770	−0.2814
EA	−0.9023	−0.7175	−0.8202	−0.0101	0.1838	0.8913	0.5948
4-hBA	0.9473	0.9998	0.9868	0.7045	0.5537	−0.3226	0.1343
*trans-p*-CA	0.8200	0.5944	0.7160	−0.1531	−0.3416	−0.9533	−0.7179
CA	0.8884	0.9885	0.9512	0.8032	0.6726	−0.1766	0.2817
FA	−0.0201	−0.3382	−0.1823	−0.9066	−0.9712	−0.7810	−0.9777
SiA	0.8863	0.9878	0.9498	0.8059	0.6760	−0.1722	0.2861
ChA	−0.5672	−0.8004	−0.6935	−0.9892	−0.9420	−0.3082	−0.7007
NeoChA	−0.8167	−0.5898	−0.7121	0.1586	0.3469	0.9550	0.7218
*o-*CA	0.9802	0.8658	0.9350	0.2537	0.0616	−0.7537	−0.3807
CiA	−0.1710	0.1524	−0.0086	0.8094	0.9078	0.8858	0.9998
EGC	−0.4830	−0.1782	−0.3342	0.5737	0.7214	0.9887	0.9515
C	0.6096	0.3247	0.4726	−0.4435	−0.6087	−1.000	−0.8942
EC	−0.3766	−0.6526	−0.5221	−0.9976	−0.9922	−0.5057	−0.8379
EGCG	0.1320	0.4415	0.2914	0.9482	0.9918	0.7060	0.9480
ECG	−0.4170	−0.6853	−0.5592	−0.9997	−0.9857	−0.4672	−0.8130
R	0.3214	0.0024	0.1632	−0.7088	−0.8320	−0.9469	−0.9908
Q	−0.9966	−0.9182	−0.9699	−0.3646	−0.1774	0.6721	0.2705

GA—gallic acid, PA—protocatechuic acid, VA—vanillic acid, SA—syringic acid, PAEE—protocatechuic acid ethyl ester, EA—ellagic acid, 4-hBA—4-hydroxy benzoic acid, *trans-p*-CA—*trans-p*-coumaric acid, CA—caffeic acid, FA—ferulic acid, SiA—sinapic acid, ChA—chlorogenic acid, NeoChA—neochlorogenic acid, *o*-CA—*o*-coumaric acid, CiA—cinnamic acid, EGC—epigallocatechin, C—catechin, EC—epicatechin, EGCG—epigallocatechin-3-gallate, ECG—epicatechin-3-gallate, R—rutin, Q—quercetin, ABTS—antioxidant activity values measured by scavenging of ABTS radicals (mg TE/g), DPPH—antioxidant activity values measured by scavenging of DPPH radicals (mg TE/g), FRAP—antioxidant activity values measured by reduction of Fe^3+^-TPZ complexes (mg TE/g), ACW—antioxidant activity of water-soluble antioxidants (AAE/g), ^1^ ACW—antioxidant activity of water-soluble antioxidants (TE/g), ACL—antioxidant activity of lipophilic antioxidants (TE/g), IAC—integral antioxidant capacity (TE/g).

**Table 6:** The relationships between several evaluation parameters of undigested part of matcha.

	Antioxidant Activity Values						
*r*	ABTS	DPPH	FRAP	ACW	^1^ ACW	ACL	IAC
Phenolics							
GA	−0.2368	−0.2088	0.0268	−0.2350	−0.3688	−0.9662	−0.9301
PA	−0.9075	−0.9192	−0.9860	−0.9082	−0.8407	0.4397	−0.1806
VA	−0.2761	−0.3035	−0.5189	−0.2778	−0.1406	0.9669	0.6256
SA	0.5514	0.5273	0.3129	0.5499	0.6614	0.8221	0.9995
PAEE	0.8486	0.8634	0.9578	0.8496	0.7674	−0.5473	0.0576
EA	−0.2414	−0.2691	−0.4879	−0.2431	−0.1050	0.9754	0.6532
4-hBA	0.7333	0.7525	0.8862	0.7346	0.6323	−0.6956	−0.1321
*trans-p*-CA	−0.5940	−0.5710	−0.3621	−0.5924	−0.6993	−0.7114	−0.9998
CA	0.6904	0.7109	0.8562	0.6917	0.5838	−0.7382	−0.1924
FA	−0.8729	−0.8586	−0.7140	−0.8720	−0.9320	−0.4689	−0.9022
SiA	0.9994	1.000	0.9732	0.9995	0.9852	−0.0555	0.5491
ChA	−0.7900	−0.7721	−0.6013	−0.7895	−0.8672	−0.5963	−0.9571
NeoChA	0.8979	0.8849	0.7507	0.8971	0.9501	0.4206	0.8776
CiA	0.9960	0.9981	0.9846	0.9961	0.9740	−0.1114	0.5013
EGC	0.7777	0.7593	0.5851	0.7765	0.8571	0.6117	0.9622
C	0.4945	0.5192	0.7055	0.4960	0.3696	−0.8797	−0.4245
EC	−0.1017	−0.1303	−0.3596	−0.1035	0.0367	0.9968	0.7537
EGCG	0.8277	0.8277	0.6512	0.8267	0.8973	0.5431	0.9359
R	−0.9374	−0.9271	−0.8130	−0.9368	−0.9766	−0.3278	−0.8253
Q	0.1404	0.1119	−0.1247	0.1386	0.2758	0.9868	0.8896

GA—gallic acid, PA—protocatechuic acid, VA—vanillic acid, SA – syringic acid, PAEE—protocatechuic acid ethyl ester, EA—ellagic acid, 4-hBA—4-hydroxy benzoic acid, *trans-p*-CA—*trans-p*-coumaric acid, CA—caffeic acid, FA—ferulic acid, SiA—sinapic acid, ChA—chlorogenic acid, NeoChA—neochlorogenic acid, CiA—cinnamic acid, EGC—epigallocatechin, C—catechin, EC—epicatechin, EGCG—epigallocatechin-3-gallate, R—rutin, Q—quercetin, ABTS—antioxidant activity values measured by scavenging of ABTS radicals (mg TE/g), DPPH—antioxidant activity values measured by scavenging of DPPH radicals (mg TE/g), FRAP- antioxidant activity values measured by reduction of Fe^3+^-TPZ complexes (mg TE/g), ACW—antioxidant activity of water-soluble antioxidants (AAE/g), ^1^ ACW—antioxidant activity of water-soluble antioxidants (TE/g), ACL—antioxidant activity of lipophilic antioxidants (TE/g), IAC—integral antioxidant capacity (TE/g).

## Data Availability

The data presented in this study are available in the article.
